# Evaluation of direct costs associated with the management of clinical stage of malaria in children under five years old in Gabon

**DOI:** 10.1186/s12936-021-03862-4

**Published:** 2021-07-30

**Authors:** Gaëtan Moukoumbi Lipenguet, Edgard Brice Ngoungou, Euloge Ibinga, Jean Engohang-Ndong, Jérôme Wittwer

**Affiliations:** 1 University of Bordeaux, Population Health of Bordeaux, Inserm U1219, EMOS team. ISPED, 146 rue Léo-Saignat, 33076 Bordeaux cedex, France; 2 Department of Epidemiology-Biostatistics and Medical Informatics (DEBIM), Faculty of Medicine, University of Health Sciences, BP: 4009 Libreville, Gabon; 3 University of Health Sciences, Research Unit in Epidemiology of Chronic Diseases and Environmental Health (UREMCSE), BP: 11587 Libreville, Gabon; 4 UMR 1094 Inserm partner IRD-Tropical Neuroepidemiology (NET), Faculty of Medicine of the University of Limoges, 87 025 Limoges, France; 5 Department of Biological Sciences, Kent State University at Tuscarawas, 330 University Dr. NE, New Philadelphia, OH 44663 USA

**Keywords:** Malaria, Management cost, Hospitalization, Direct cost, Pediatric

## Abstract

**Background:**

Malaria is one of the leading causes of morbidity and mortality in African countries. It is one of the leading causes of hospital visits and hospitalization in pediatric wards for children under 5 years old. Interestingly however, the economic burden of this disease remains unknown in these endemic countries including Gabon. The purpose of this study is to assess the direct hospital cost for the management of malaria in children under 5 years old at the Libreville University Hospital Centre (CHUL, Centre Hospitalier Universitaire de Libreville) in Gabon.

**Methods:**

This research work is a retrospective study using a comprehensive review of medical records of patients seen at the CHUL over a two-year period extending from January 2018 through December 2019. The study focused on children under 5 years old, admitted for malaria in the paediatric ward of the CHUL. The analysis targeted specifically direct hospital costs, which excluded salary and wages of health care workers. The monetary currency used in this study was the CFA francs, as that currency is the one used in Central Africa (as reference, 1 Euro = 656 CFA francs).

**Results:**

For the set timeframe, 778 patient records matched the study criteria. Thus, out of 778 admitted patients, 58.4% were male while 41.5% were female. Overall, the average age was 13.2 months (± 13.8 months). The total cost incurred by the hospital for the management of these 778 malaria patients was 94,922,925 CFA francs (144,699.58 €), for an average expense per patient topping at 122,008 CFA francs (185.99 €). The highest expenditure items were hospitalizations (44,200,000 CFA francs, 67,378.1 €), followed by drugs (26,394,425 CFA francs, 40,235.4 €) and biomedical examinations (14,036,000 CFA francs, 21,396.34 €).

**Conclusion:**

The financial burden for managing malaria in the paediatric ward seems to be very high, not only for the hospital, but also for families in spite of the government medical insurance coverage in some cases. These findings bring new insights as to the urgency to develop policies that foster preventive initiatives over curative approaches in the management of malaria in children in endemic countries.

## Background

Malaria has remained a burden for African countries for years. The World Health Organization [1], in its 2018 malaria progress report, estimated the number of cases at 219 million, 92% of which were in African countries. In 2019, this number increased to 229 million of the malaria cases, for an estimated total of 409,000 deaths, according to the WHO statement [2]. The world records about one million deaths each year due to malaria and among which 91% are reported in Africa, with 85% of that toll representing children under 5 years [2]. In Gabon, in 2019 malaria ranked first among the top ten causes of morbidity and mortality [3]. All ages combined; the total number of malaria cases recorded was 124,478 cases. For the same period, the number of cases of children aged 0–5 years was estimated at 41,087 cases in health care facilities. In spite of the fact that deaths are under-reported, 120 deaths were nevertheless recorded for the year 2019 in the main health care facilities in the country [2].

Management of this disease involves significant logistics for hospitals and substantial financial resources for the government, patients or families of patients. In its financial report for the elimination of malaria, the WHO declared a funding of US$ 3 billion in 2019, a fund considered insufficient [2]. In countries where malaria remains endemic, very few studies have been conducted to evaluate the direct cost of treating this disease [4]. At this point in time, there is no known studies evaluating the economic burden of malaria management in Gabon, which is one of the African countries with a high incidence of malaria.

Thus, the aim of this study was to assess the direct hospital cost of the management of malaria in children under 5 years old at the Libreville University Hospital Centre (CHUL, Centre Hospitalier Universitaire de Libreville) in Gabon. The findings of this study could provide a basis for further investigation on the cost-effectiveness of prevention programmes and eventually to the development of prevention policies that could be beneficial to governments and families of patients in endemic regions.

There is very little data on the financial burden of malaria on Gabon’s health system and households. A few studies have been conducted in African countries, but not enough to clearly assess the economic burden of the endemic [5–7]. This study modestly aims to help fill this gap. The results will be discussed and compared with those of other studies conducted to assess the burden of this disease.

## Methods

The study took place in the paediatric department of the Centre Hospitalier Universitaire de Libreville (CHUL) in Gabon. In Gabon, the CHUL was a hospital of choice to conduct this study because in one hand, that hospital has the largest paediatric ward in the country, on another hand, the facility is adequately equipped to care for children and very importantly, to keep accurate records of medical procedures of patients admitted in the facility. Thus, a retrospective study was performed based on the review of patient records over a two-year period from January 2018 to December 2019. The retrospective study started in 2018 because the implementation of the National Health Information System (SNIS, Système National d’Information Sanitaire) did not take place in Gabon before then. The SNIS helped Gabon create a centralized health information system that allowed the CHUL to store and keep accurate information of patients seen in the facility. Hence, no accurate health information could be obtained before that year. So, data were collected retrospectively on the basis of patient records archived in the paediatric department on one hand and using the nomenclature of procedures and the list of drugs prescribed on the other hand for the cost of procedures. All files available onsite were taken into account.

The first step of this study was to collect clinical and biological parameters necessary for the diagnosis of malaria at admission using a pre-established survey form. This was followed by examining and sorting the files according to the target age of admitted patients (0 to 5 years). After the files were examined, the form was filled out. More specifically, the socio-demographic data of children, the examinations prescribed to diagnose and monitor evolution of the disease, and medications administered during hospitalization were recorded on the form. The second step consisted in determining the unit costs of all the procedures executed and the costs of hospital stays.

The monetary assessment of the pharmaceutical products used (drugs and medical devices) was made on the basis of the repository of medicines covered by the National Health Insurance and Social Guarantee Fund [8, 9]. The National Health Insurance and Social Guarantee Fund (CNAMGS) is a government-owned and government-managed compulsory health insurance and social guarantee entity designed to assist the Gabonese population with their health expenses. It is the only government insurance that covers all Gabonese citizens who are duly registered and maintain their membership in good standing. It covers health care prescriptions and procedures according to a percentage established by the government. Typically, the percentage of coverage varies between 50% and 80% depending on the medical procedure, which could be a clinical examination, a laboratory test, an imaging examination, and/or a drug prescription. The rest of the balance is covered by the patient and/or their family. In health care facilities, that balance due by the patient is called moderator ticket or co-payment. In this study, the costs for medical products not reimbursed by the government insurance were obtained in pharmacies. Although private insurances exist in Gabon, there is no complementarity between them and the government health insurance.

Out-of-pocket expenses for insured patients or co-payments were evaluated in relation to the level of coverages set by the health insurance for different health procedures and medications prescribed.

The public health care is set in Gabon such that public hospitals are completely supported by the government. In other words, the costs for the maintenance and operation of the premises are covered by the government budget regardless of the frequency use by patients. Furthermore, salaries and wages of health professionals and allies are paid by the government and do not involve in any way a contribution from the patients. Gabonese health workers are civil servants whose salaries and wages are fixed regardless of the number of patients seen or medical procedures executed. Therefore, the costs pertaining to the maintenance, management of the health care facility and salary and wages were not included as these are all considered indirect cost. However, hospitalization costs which included bed occupancy, food and beverages consumed by patients were all part of direct costs associated with malaria management.

The study performed here was primarily observational, retrospective, and based exclusively on medical records. It was non-invasive and carried no physical, mental, nor emotional risk to the patient. It did not involve human or animal experimentation. Therefore, this study was exempt from ethics approval as this is the procedure currently applied in Gabon. Nevertheless, in compliance with the research protocols in Gabon, two research authorizations were obtained for the collection of information on medical records. One was provided by the General Secretary of the Ministry of Health (authorization N°002395/MS/SG/PSNIS) and the second one was issued by the Chief Executive Officer of the CHUL (authorization N°0078/MS/CHUL/DG/DGA).

All data were recorded in a database created using an Excel spreadsheet. Data collected and compiled in the database were analysed with the R software (version 3.6.1). Quantitative variables were expressed in means with their standard deviation while qualitative data were expressed in percentage values.

## Results

The study involved 778 patients who were admitted to the paediatric ward during the designated period. Analysis of all cases documented revealed that 58.5% were male and 41.5% female. Furthermore, analysis of data showed that the overall average age of patients was 13.2 months (± 13.8 months). The median age in the female group was 7 months of age while in the male group, it was 9 months. Also, the screening of patient files showed that more than half of them (58.5%) had an age range that varied between 1 month and 12 months old and that 51.7% (402) of patients did not have any health insurance coverage. 488 patients exhibited complications among which 28% suffered of anaemia and 16% had prostration. Table 1 summarizes the socio-demographic characteristics and complications recorded among patients.

**Table 1:** Sociodemographic characteristics and complications due to malaria at the time of admission

Characteristics	Patient set N = 778	Insured patient N = 376 (48.3%)	Uninsured patient N = 402 (51.7%)
Age in months, average (SD)	13.2 (± 13.8)	14.0 (± 15.0)	12.4 (± 12.6)
Gender, N (%)			
Male	455 (58.5)	224 (59.6)	231 (57.5)
Female	323 (41.5)	152 (40.4)	171 (42.5)
Age range in months, N (%)			
[1–12]	489 (62.9)	235 (62.5)	254 (63.1)
[12–24]	185 (23.9)	83 (22.1)	102 (25.4)
[24–36]	57 (7.3)	26 (6.9)	31 (7.7)
36–48]	33 (4.2)	23 (6.1)	10 (2.5)
[48–60]	14 (1.8)	9 (2.4)	5 (1.2)
Complications, N (%)			
None	290 (37.3)	137 (36.4)	155 (38.5)
Consciousness disorders	9 (1.2)	7 (1.9)	2 (0.2)
Convulsion	62 (7.9)	24 (6.4)	38 (9.4)
Prostration	126 (16.2)	59 (15.7)	67 16.7)
Respiratory distress	29 (3.7)	17 (4.5)	12 (2.9)
Jaundice	32 (4.1)	18 (4.8)	14 (3.5)
Bleeding	5 (0.6)	5 (1.3)	0 (−)
Anaemia	225 (28.9)	111 (29.5)	114 (28.4)

There was no significant variation in average expenditure by socio-demographic characteristics, between age groups or even by gender. The age group with the highest average cost was the 24 to 36 month-old group which spent an average of 122,975 CFA francs (187 €), followed by the 12 to 24 month-old group with an average expenditure of 122,711 CFA francs (187 €).

By gender, expenses incurred by male patients were higher (125,253 CFA francs, 191 €) average than those of female patients (117,438 CFA francs, 179 €). The age group with the highest average out of pocket expense, meaning the remaining balance after insurance coverage, was the 1 to 12 month-old group which showed a remaining balance of 28,203 CFA francs (43 €) due by the patient, followed by the 24 to 36 month-old group with a remaining balance of 27,983 CFA francs (42 €) (Table 2).

**Table 2:** Average costs of malaria cases by socio-demographic characteristics

Characteristics	Uninsured patients	Insured patients		
		Total expenditure	Insurance coverage	Co-payment
Age in months				
[1–12]	115,686 (44,349)	129,500 (66,994)	101,297 (53,536)	28,203 (15,364)
[12–24]	125,217 (61,178)	119,631 (58,738)	92,520 (45,093)	27,111 (18,482)
[24–36]	116,530 (43,646)	136,530 (87,878)	102,677 (70,389)	27,983 (17,639)
[36–48]	130,842 (66,883)	111,998 (34,680)	88,007 (27,593)	23,990 (7,499)
[48–60]	110,357 (29,305)	106,507 (27,811)	83,795 (22,301)	22,712 (5,689)
Gender				
Male	129,851 (54,883)	129,851 (73,715)	101,596 (58,893)	28,254 (16,622)
Female	115,354 (41,257)	119,783 (48,324)	93,252 (37,465)	26,531 (14,389)

The total expenditure for the care of the study population was 94,922,925 CFA francs (144,699 €) which represented an average of 122,008 CFA francs (186 €) per patient. Uninsured patients incurred an average total expenditure of 118,480 CFA francs (180 €). After insurance coverage of expenses, the remaining balance due by insured patients was found to be 27,558 CFA francs (42 €) per patient from a total pre-insurance coverage cost of 10,361,901 CFA francs (15,795 €). Health insurance reimbursement to healthcare service facilities for patient care were determined to cost of 36,931,352 CFA francs (56,298 €) overall.

Of the overall cost caused by malaria, the highest expenditure item was associated with patient hospitalization, at an overall cost of 44,200,000 CFA francs (67,378 €) which represented 46.56% of the total cost of malaria patient care during the study period. Far behind hospitalization cost was the cost amount associated with the purchase of malaria medicines at a grand total of 26,394,425 CFA francs (40,235 €) representing 27.8% of the total cost of malaria management cases for the period under study (Table 3).

**Table 3:** Average costs by expenditure item

Type of expenditure	Global cost (%)	Uninsured patients	Insurance			Overall Average (N = 778)	Extremes
		Average (N = 402)	Insured patients (N = 376)	Patient responsibility (N = 376)	Insurance coverage (N = 376)		
Medical visit	7,780,000 (8.2)	10,000	10,000	2000	8000	10,000	[0, 0–10]
Hospitalization	44,200,000 (46.6)	53,681	60,159	12,096	48,385	56,812	[0, 0–480]
Laboratory tests	14,036,000 (14.8)	18,044	18,037	3728	14,308	18,041	[0–30, 500]
Imaging exams	2,512,500 (2.6)	2779	3710	1484	2226	3229	[0–7, 500]
Drugs	26,394,425 (27.8)	33,974	33,874	8313	25,561	33,926	[3960–177,271]
Total	94,922,925 (100.0)	118,480	125,781	27,558	98,223	122,008	[39,073–531,740]

Of the 778 malaria patient records reviewed, data analysis showed that 335 patients developed pulmonary oedema as a result of malaria-related complications. The average cost associated with the care of these particular patients was 137,087 CFA francs (209 €). In this particular instance, there was no significant difference in the costs incurred when comparing insured and uninsured patients.

The malaria cases record indicated that the most common laboratory examinations performed as part of the diagnosis were blood smears, complete blood counts, blood urea nitrogen tests, and creatinine tests. Of all the malaria records screened during the study, 93.3% included blood smears as a diagnosis test, and 97% contained the results of complete blood counts. Furthermore, blood urea nitrogen tests were performed in 60.4% of the cases analysed while creatinine tests were done in 59.5% of the cases included in this study (Table 4).

**Table 4:** Unit costs per test and average cost by test type and per patient

Type of test	Number of tests (%)	Unit cost	Global cost	Insured patients	Uninsured patients
Imaging exam					
Pulmonary edema	335 (43.1)	7500	137,087	136,200	138,194
Laboratory tests					
Blood smear	726 (93.3)	3000	122,308	125,503	119,233
Complete blood count	755 (97.0)	7500	122,516	125,883	119,332
Blood urea nitrogen	470 (60.4)	3500	125,704	129,500	122,065
Creatine	463 (59.5)	3500	125,339	129,595	121,316
Transaminase	351 (45.1)	6000	126,204	127,690	124,824
Blood glucose	242 (31.1)	3500	130,688	131,339	130,089
Total protein	9 (1.2)	3500	114,628	116,083	113,900
Tck	3 (0.4)	3500	75,336	59,110	83,450
Parasitaemia	2 (0.3)	3000	76,920	76,920	–

## Discussion

The purpose of this study was to assess the direct hospital cost of malaria management in children, whose ages ranged between 0 and 5 years old. The cost analysis was mainly based on costs incurred by the government health insurance coverage and the amount of money due to be paid by patients whether they were covered by health insurance or not. However, analysis of the costs did not include the compensation healthcare workers.

This study showed a cost of malaria management per patient of 122,008 CFA francs (186 €), meaning a total expenditure of 95,083,524 CFA francs (144,944 €) for a total of 778 malaria patients whose age varied between 0 and 5 years. Uninsured patients registered an average expenditure four times higher than that observed in patients receiving health benefits from the government insurance scheme. On average, uninsured families spent 118,480 CFA francs (181 €) to care for their sick child. The expense incurred by these families was sadly very close to the monthly national minimum wage of 150,000 CFA francs (229 €), reflecting the heavy and costly burden an episode of malaria leading to child’s hospitalization for uninsured families. In fact, the expenses incurred by uninsured families represented up to 78% of the national minimum wage. Conversely, families receiving health coverage from the government incurred a much lower out-of-pocket expense averaging 27,558 CFA francs (42 €). A closer look at malaria management costs for insured families shows that the health insurance policy covers 80% of costs, while insured families themselves paid only 20% of the cost. While this is still a great deal for insured families, the cost also represents a major expense for the government-provided health insurance coverage. Indeed, the government health insurance paid to healthcare facilities an average of 98,223 CFA francs (150 €) per patient, which shows the onerous cost of clinical phase of malaria management for the government.

While there has been little work in the literature on the actual cost of malaria management, with the majority of studies focusing on management of clinical cases of malaria in adult patients, the financial burden of malaria is extremely high in many countries where malaria is endemic. This is the case, for instance, in the study conducted by Faye et al. [10], in which the authors performed the economic evaluation of rapid diagnostic tests in the treatment of malaria in Ziguinchor, Senegal. The study involved 379 cases of malaria and included patients of all ages. That study reported an overall cost of clinical malaria management of 299,957 CFA francs (457 €). The financial cost for patients was 184,500 CFA francs (281 €) while the government health insurance covered 115,457 CFA francs (176 €). Although the overall cost of 299,957 CFA francs (457 €) seems lower than the findings in the present study, Faye and his colleagues considered the costs in their study to be too high given the lower count of their population. Interestingly however, the out-of-pocket expenses paid by insured patients were much higher in their study than in the current study, confirming the tremendous economic burden of clinical management of malaria for governments and families.

In a study published in 2005 by Forlack et al. [11], which examined the cost-effectiveness of managing severe malaria in children in two district hospitals in Cameroon, the costs of managing malaria were much lower than those observed in this analysis. Indeed, in their study, the hospital cost for each episode of severe malaria ranged from 26,000 to 36,000 CFA francs (40 to 55 €). Their study showed that the overall direct costs, which included costs incurred before and during hospitalization ranged from 27,000 to 39,000 CFA francs (41 to 59 €). While the financial impact reported in Cameroon was much lower than that shown in this study performed in Gabon, the difference may be accounted for by the difference in the cost of living in the two countries which is much higher in Gabon than Cameroon. Thus, with a national minimum wage of 36,000 CFA francs (55€) in Cameroon, the cost of malaria management of 39,000 CFA francs (59 €) is extremely high for families, which reinforces the assessment of the heavy burden experienced by populations in the management of malaria in healthcare facilities in both Cameroon and Gabon. Similarly, in their study performed in Ivory Coast, Kouadio et al. [12] estimated an average direct cost per case of malaria around 8,725 CFA francs (13.3 €). While this cost appears much lower than that observed in other studies, including this one, it is nevertheless perceived by the authors as a heavy economic burden for low-income households living in poor neighbourhoods of Abidjan.

The highest expenditures in this study were hospitalizations and drug prescriptions which accounted for 74.4% of total expenses. These findings are similar to those reported by Couitchéré et al. [13] in Ivory Coast, where the report explains that the highest costs of malaria care are associated with paraclinical examinations, drug prescriptions, and hospitalizations.

The cost of treating malaria represents a heavy burden for countries with high malaria prevalence. This heavy burden is paid by uninsured families, who sometimes resort to borrowing money for the care of their relatives. While, this study did not highlight these aspects because a population survey was not conducted, nevertheless, other studies conducted among households have highlighted the difficulties faced by these families. In the study on the financing of the management of severe malaria in children by households in Kinshasa, Ilunga-Ilunga et al. [14] examined the financial protocol used by families to pay for expenses incurred during malaria episodes. They reported in their study that 56% of households turned to external financial sources, including selling goods, borrowing money, and putting in pawn some valuable assets to care for their hospitalized children. Furthermore, Adhikari et al. [15], in their study of treatment-seeking behaviour for febrile illnesses and its implications for malaria control and elimination in the Savannakhet province in Laos, reached similar conclusions. This province of Laos is landlocked and poor, making it difficult for people to use health services due to lack of money. The first recourse for disease treatment in that region was spiritual healers, a solution considered more economical by these populations. The situations highlighted in these studies support the idea about the burden of malaria management in endemic countries.

The present study is not without limitations. Indeed, this study does not take into account the indirect costs related to the salaries of health professionals, patient transport and the loss of income of accompanying persons. Health professionals are civil servants, and their salaries are paid by the government budget. In spite of multiple attempts, the research team was not able to obtain this information, which is inaccessible to the public.

## Conclusion

Malaria remains one of the leading causes of morbidity and mortality, particularly among children. The management of complicated cases generates considerable expenses and is a burden for patients' families and the government. The financial burden is not only heavy for families without social security coverage, but also for the government which bears the greatest part of the financial burden generated by insured patients and their families. Indeed, in Gabon, the health insurance offered by the government covers up to 80% of the costs incurred for the management of malaria cases for qualified families. The results of this study show that the direct cost of hospital care is very high in a country where a large proportion of the population lives below the poverty line. These results highlight the urgent need to strengthen and develop public health policies that would favour prevention as a major mean of controlling and fighting malaria, not only in Gabon, but also in all other endemic countries. A short-term and less costly solution could be to invest in the use of impregnated bed nets.

## Data Availability

The datasets used and/or analyzed for the current study are available from the corresponding author on reasonable request.
